# Editorial for the Special Issue “Medical Data Processing and Analysis—2nd Edition”

**DOI:** 10.3390/diagnostics15091170

**Published:** 2025-05-04

**Authors:** Wan Azani Mustafa, Hiam Alquran

**Affiliations:** 1 Faculty of Electrical Engineering & Technology, Campus Pauh Putra, Universiti Malaysia Perlis, Arau 02600, Malaysia; 2 Advanced Computing (AdvComp), Centre of Excellence (CoE), Universiti Malaysia Perlis, Arau 02600, Malaysia; 3 Department of Biomedical Systems and Informatics Engineering, Yarmouk University, Irbid 21163, Jordan; heyam.q@yu.edu.jo

Medical data processing and analysis have become central to advancements in healthcare, driven largely by the need for accurate diagnosis, personalized treatment, and efficient healthcare system management [1,2,3]. A major trend in this domain is addressing the limitations of insufficient quality data through human-in-the-loop (HITL) approaches [4], explainable AI (XAI) frameworks [5], and blockchain-based security models [6]. Current expansions in machine learning, computer vision, and edge computing have significantly impacted the early detection and diagnosis of various types of diseases [7,8,9,10,11]. Many studies demonstrate innovative applicable approaches to neurological disorders, infectious diseases, developmental conditions, and medical image retrieval.

On the other hand, Tur [12] introduced a multi-modal machine learning framework for identifying COVID-19 utilizing a combination of many biochemical biomarkers and chest X-ray imaging. The proposed hybrid model combines data from both sources, and ensemble learning was employed to enhance its diagnostic accuracy over single-modality approaches. The study emphasized the impact of multimodal data fusion when concentrating on heterogeneous manifestations of infectious diseases. However, Jeon et al. [13] surveyed machine learning and explainable AI (XAI) for the dependable diagnosis of autism spectrum disorder (ASD) in pediatric patients. They developed neurodevelopmental features and behavioral assessments for building a high-performance diagnostic model. Prominently, utilizing XAI modified the interpretation, and the assessments were shown to be appropriate for clinical adoption by enhancing trustable models. In contrast, Gasmi et al. [14] investigated the enhancement of medical image retrieval by incorporating Unified Medical Language System (UMLS) concepts with a convolutional neural network (CNN)-based text indexing method. Their proposed system develops semantics that recognize medical reports and can combine these semantics with related images. Their recommended approach fills the gap between clinical language and image databases, assisting image retrieval precisely in clinical decision support systems.

Mosqueira-Rey et al. [15] emphasized the role of HITL in overcoming data bottlenecks in deep learning models for pancreatic cancer treatment, combining synthetic data augmentation using generative adversarial networks (GANs) and expert feedback through active learning. Together, these works highlight an increasing focus on privacy-preserving models and patient acceptance, though obstacles such as encryption overheads and equitable technology access persist. Singh and Mantri [16] similarly focused on improving preprocessing and feature selection strategies via Rough Set Theory (RST) to optimize data dimensionality, enhancing the performance of clinical decision support systems (CDSS). Hussain and Mishra [17] demonstrated the application of AI and big data in COVID-19 diagnosis, employing deep learning on medical images amid data scarcity challenges. Collectively, these studies underscore that while data augmentation and expert integration help mitigate data limitations, complexities in model integration and data quality assurance remain persistent issues. Parallel to data-centric innovations, patient trust and data security have emerged as critical considerations. Paccoud et al. [18] explored patient perspectives on adopting digital medical devices (DMDs) for Parkinson’s disease management, finding a general willingness tempered by demographic differences. Addressing confidentiality concerns, Affum and Enchill [19] introduced a division-free gradient descent multivariate regression approach to encrypted medical data, ensuring secure machine learning without exposing sensitive patient information.

Advancements in electronic medical record (EMR) processing have also demonstrated strong potential but revealed dependencies on robust feature extraction and explainable model structures. Pham et al. [20] developed a validated case definition for rheumatoid arthritis (RA) using a tree-based ensemble, identifying XGBoost as particularly effective for generating interpretable outcomes suitable for primary care. Singh and Mantri [16] achieved improved classification for diseases like breast cancer and thyroid disorders through integrated machine learning models and advanced feature selection. Hussain and Mishra [17] similarly used convolutional neural networks and transfer learning to enhance COVID-19 diagnosis accuracy. Although these techniques produced promising results, their reliance on structured datasets limits their real-world applicability, where data heterogeneity is common. Alongside technical challenges, interpretability and ethical concerns have come to the forefront. Hakkoum et al. [21] conducted a comprehensive evaluation of interpretability techniques for supervised machine learning models, emphasizing the tension between model transparency and performance in medical applications. Hossain et al. echoed this concern, noting healthcare professionals’ hesitancy to adopt deep neural networks (DNNs) due to their “black-box” nature, thus advocating for stronger development of XAI methods. Shakhovska et al. [22] proposed a hybrid XAI system combining multiple neural network architectures to improve interpretability and clinical relevance, suggesting a promising avenue toward balancing performance and transparency.

Ethical challenges in AI-driven healthcare also involve the heavy reliance on statistical generalizations rather than individualized patient evidence. Holm [23] argued that AI systems based solely on statistical estimations can lead to ethical dilemmas in resource allocation, resonating with critiques from Hakkoum et al. [21] and Hossain et al. [24] regarding the need for more trustworthy, context-sensitive AI models. While statistical models may provide transparency, they often lack the nuanced sensitivity to handle complex biological variations, highlighting a need for future models that integrate statistical clarity with deep learning capabilities.

From a practical application standpoint, machine learning has been increasingly utilized to predict healthcare outcomes and optimize system behaviors. Park et al. [25] illustrated this by predicting the closure of medical and dental clinics using administrative health insurance data combined with Support Vector Machines (SVMs), Random Forests (RF), and Extreme Gradient Boosting (XGBoost). Similarly, Maheshwari et al. [26] applied machine learning and blockchain technologies to predict transaction types within healthcare systems, advancing decentralized healthcare data management. Security, data integrity, and system optimization have become key priorities in handling decentralized medical data. Khan et al. [27] highlighted the importance of blockchain in facilitating secure communications among heterogeneous medical devices integrated through AI-enabled machine learning models.

Karthiyayini et al. [28] addressed challenges in the secure transmission of medical image data by proposing the Enhanced Model for Medical Image Data Security (EM-MIDS), incorporating machine learning approaches such as K-Means Clustering (KMC) and Random Forest (RF) for classification, supported by Support Vector Machine (SVM) for security evaluation. The integration of chaotic maps and channel-based methodologies added robustness to the encryption–decryption framework. Comparative studies suggested EM-MIDS’s superiority over existing models, particularly concerning data accuracy and privacy integrity. Meanwhile, Alyahyan [29] introduced a Transformer-based Attention-Guided CNN (TAGCNN) for disease diagnosis, achieving notable improvements in accuracy compared to conventional models like ResNet50, AlexNet, and DenseNet169. While both studies demonstrated the growing efficacy of machine learning in medical imaging, a potential limitation is the narrow focus on specific datasets, such as benchmark medical images or osteoporosis X-rays, which may limit generalizability across broader clinical applications.

Machine learning’s application in triaging outpatient care through heterogeneous data further illustrates the transformative potential of automated systems. Salman et al. [30] developed an early triage prediction model using multiple machine learning algorithms, such as SVM, Random Forest, Decision Tree, Logistic Regression, Naive Bayes, and K-Nearest Neighbor, with Decision Tree algorithms achieving the highest performance of 93.5%. That study emphasized the importance of integrating diverse Internet of Medical Things (IoMT) data, including ECG, blood pressure, and SpO2 measurements, into patient assessment processes. Complementing this, Kural et al. [31] explored the application of supervised and unsupervised learning methods like Sammon mapping and Extreme Gradient Boosting (XGBoost) for identifying anaphylaxis in claims databases. Their findings highlighted the value of automated feature selection pipelines for refining disease identification algorithms, particularly in settings where ground truth data are sparse or varied. However, both research efforts acknowledged the complexity of handling heterogeneous medical datasets, often complicated by inconsistencies in data quality and format, underlining a need for more robust, adaptable models capable of handling real-world clinical variability. The challenge of generalization in artificial intelligence (AI) models for healthcare remains significant, particularly when confronting biases intrinsic to training datasets. Ong Ly et al. [32] systematically investigated shortcut learning, where models inadvertently learn irrelevant patterns from biased data acquisition processes rather than genuine clinical features. Their development of PEst, a bias-corrected external accuracy estimator, significantly improved generalization assessments without requiring external datasets, reducing overestimation of model performance by an average of 20%.

In conclusion, medical data processing and analysis are rapidly advancing through human-in-the-loop approaches, privacy-preserving AI, blockchain-based security, and explainable machine learning frameworks. However, the field must address crucial challenges related to model generalizability, ethical considerations, patient trust, equitable access, and computational efficiency. Future research must prioritize interdisciplinary strategies that integrate technical innovation with patient-centered, transparent, and secure healthcare solutions to ensure AI-driven tools are both accurate and ethically sound.
